# Communication Efficiency and Congestion of Signal Traffic in Large-Scale Brain Networks

**DOI:** 10.1371/journal.pcbi.1003427

**Published:** 2014-01-09

**Authors:** Bratislav Mišić, Olaf Sporns, Anthony R. McIntosh

**Affiliations:** 1 Rotman Research Institute, Baycrest Centre, Toronto, Canada; 2 Department of Psychology, University of Toronto, Toronto, Canada; 3 Department of Psychological and Brain Sciences, Indiana University, Bloomington, Indiana, United States of America; University of California, Santa Barbara, United States of America

## Abstract

The complex connectivity of the cerebral cortex suggests that inter-regional communication is a primary function. Using computational modeling, we show that anatomical connectivity may be a major determinant for global information flow in brain networks. A macaque brain network was implemented as a communication network in which signal units flowed between grey matter nodes along white matter paths. Compared to degree-matched surrogate networks, information flow on the macaque brain network was characterized by higher loss rates, faster transit times and lower throughput, suggesting that neural connectivity may be optimized for speed rather than fidelity. Much of global communication was mediated by a “rich club” of hub regions: a sub-graph comprised of high-degree nodes that are more densely interconnected with each other than predicted by chance. First, macaque communication patterns most closely resembled those observed for a synthetic rich club network, but were less similar to those seen in a synthetic small world network, suggesting that the former is a more fundamental feature of brain network topology. Second, rich club regions attracted the most signal traffic and likewise, connections between rich club regions carried more traffic than connections between non-rich club regions. Third, a number of rich club regions were significantly under-congested, suggesting that macaque connectivity actively shapes information flow, funneling traffic towards some nodes and away from others. Together, our results indicate a critical role of the rich club of hub nodes in dynamic aspects of global brain communication.

## Author Summary

A fundamental question in systems neuroscience is how the structural connectivity of the cerebral cortex shapes global communication. Here, using computational modeling in conjunction with an anatomically realistic structural network, we show that cortico-cortical communication is constrained by high-level features of brain network topology. We find that neural network topology is configured in a way that prioritizes speed of information flow over reliability and total throughput. The defining characteristic of the information processing architecture of the network is a densely interconnected rich club of hub nodes. Namely, rich club nodes and connections between rich club nodes absorb the greatest proportion of total signal traffic. In addition, rich club connectivity appears to actively shape information flow, whereby signal traffic is biased towards some nodes and away from others. Finally, synthetic networks containing a rich club could almost perfectly reproduce the information flow patterns of the real anatomical network. Altogether, our data demonstrate that a central collective of highly interconnected hubs serves to facilitate cortico-cortical communication. By simulating communication on a static structural network we have revealed a dynamic aspect of the global information processing architecture and the critical role played by the rich club of hub nodes.

## Introduction

Constrained by finite resources, such as metabolism and physical space, which place severe limits on the number and density of synaptic connections, brain networks are an example of how optimized topology may facilitate information flow. The structural topology of cortical networks can be represented and formally studied using the graph model [1]–[3], whereby the brain is spatially parcellated into a set of grey matter nodes interconnected by a set of white matter edges [4], [5]. This approach has revealed several aspects of network organization that theoretically confer an increased capacity for information processing, including small-world connectivity [6]–[8], the presence of hubs [9] and cores [10], cost-efficient spatial embedding [11], [12] and the coexistence of local segregation and global integration [13].

Recent studies have also uncovered a “rich club” of hub nodes that are more densely interconnected with each other than predicted by chance [14], [15] and that participate in a disproportionately high number of shortest paths in the network [16], [17]. The rich club is hypothesized to act as a central backbone for signal traffic, allowing for rapid integration and dissemination of signal traffic [16].

While this graph theoretic approach can articulate the diverse properties of static neural connectivity, it does not take into account the dynamics of information flow on that connectivity. If information flow is introduced into the network, how does neural connectivity influence the efficacy and speed of communication? In other words, how does network topology enable and constrain the capacity of brain networks to globally integrate information? For instance, while certain areas may bridge distant communities and potentially function as hubs by virtue of their connectivity, other areas may be ill-suited as conduits for information transfer because of their position in the network. Under conditions of elevated network traffic such regions could become bottlenecks, imposing limits on the relay of information [18].

**Figure 1:**
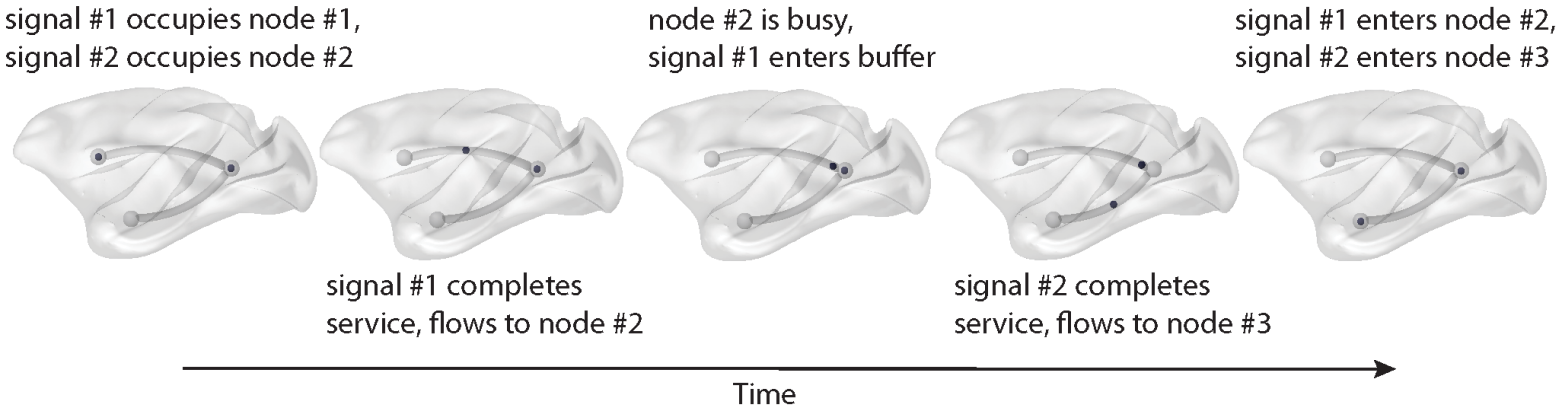
Discrete-event simulation. Schematic showing the propagation of two signal units in a simple 3-node, 2-pathway network.

To determine the effect of topology on inter-regional communication, we implemented a macaque anatomical brain network as a communication system in which units of information flow between grey matter nodes along existing anatomical paths (Fig. 1). This allowed us to estimate several metrics of information flow in the network, including the proportion of time a given brain region is in use (utilization), the load on a given brain region (node contents), the time it takes for a unit of information to travel from its source region to its target region (transit time) and the probability of losing information (blocking). The goal of the present study was to use these performance metrics to address the following questions about communication in brain networks. First, does the unique topology of brain networks offer any particular advantage in terms of information processing, and how do brain networks compare to other networks with the same number of nodes and edges, but different topologies? Second, which features of brain network organization contribute most to its capacity for efficient communication? Third, which anatomical regions and pathways are most important for communication?

## Results

### Network statistics

Information flow on the network was simulated by generating signal units with specific, randomly-selected source and destination nodes. Each signal unit diffused to one of the neighbouring nodes, until it reached its destination. If a signal unit arrived at a node that was occupied, a queue was formed. A maximum buffer size was imposed, such that a signal unit arriving at a full buffer caused the oldest signal unit in the queue to be removed from the system. A signal unit was removed from the network once it reached its destination node. Simulation intensity was controlled parametrically to investigate the effect of increasing load on communication efficiency.

Measures of information flow on the macaque network were compared against a spectrum of degree-matched control networks with an equal number of nodes and edges, but systematically altered topology. One set was comprised of randomized networks, while the other set was comprised of latticized networks (see Materials and Methods for more information on how surrogate networks are generated). Under conditions of increasing load (see SI Fig. S1), all networks experienced increased blocking and utilization, as well as decreased throughput, thus exhibiting signs of congestion. Mean transit times for signal units that reached their destination also decreased with increasing load, but this counterintuitive observation is the result of decreased throughput. At lower network load, more signals reach their destination but some may take a long time to do so, which increases the mean transit time. As the network becomes congested, many such signals may get dropped at over-utilized nodes and never reach their destination, and thus cannot influence the mean transit time.

**Figure 2:**
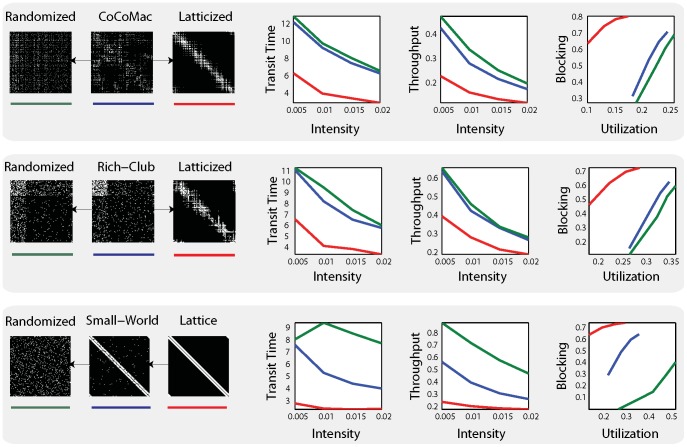
System-level statistics. Simulations were run for three different scenarios: CoCoMac brain (top), small-world network (middle) and rich-club network (bottom) and their respective randomized (green) and latticized (red) control networks. For each network, the mean transit time, utilization, blocking probability and throughput are plotted at four different intensities. For the starting networks in each scenario (CoCoMac, lattice and rich-club), the curves represent the average over 100 simulations. For the randomized/latticized versions of each network, the curves represent the average across 100 simulations on 100 realizations of each network.

The macaque network was intermediate on all information flow statistics compared to the randomized and latticized networks. This is consistent with the fact that the randomized and latticized networks represent two extreme and diametrically opposite network configurations and suggests that the organization of the macaque network serves to strike a balance between speed, reliability, utilization and total throughput. Compared to its randomized control network, information flow on the macaque network was characterized by significantly higher loss rates, faster transit times and lower throughput (Fig. 2 top, 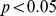 for all measures, Tables S2,3,4), suggesting that neural connectivity may be optimized for speed rather than fidelity. In general however, the two networks performed similarly, while the latticized control network performed much differently, with significantly lower utilization and throughput, shorter transit times and near total loss of information (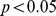 for all measures).

We next sought to determine which feature of network topology most contributed to this pattern of results. To investigate the degree to which the existence of a rich club influences information flow, a synthetic network containing a rich club was created, as well as a set of degree-matched randomized and latticized surrogate networks (Text S1, Section 7, Fig. S7). Information flow on these networks was contrasted with a canonical small world network, which is a ubiquitous and well-studied model for many different kinds of information-processing networks, including neural networks [19]. The pattern of results produced by the synthetic small world and rich club networks and their respective randomized and latticized surrogate networks were considerably different (Fig. 2, middle and bottom). Importantly, the system statistics associated with the rich club network were nearly identical to the macaque network (see Text S1, Section 4, Table S1). The results observed for the macaque network were also similar to the canonical Watts-Strogatz small world network [19], but to a significantly lesser extent (Text S1, Section 4, Table S1). Overall, this suggests that the rich club is an important topological feature for information flow in the brain, as defined by these four statistics.

### Node statistics

**Figure 3:**
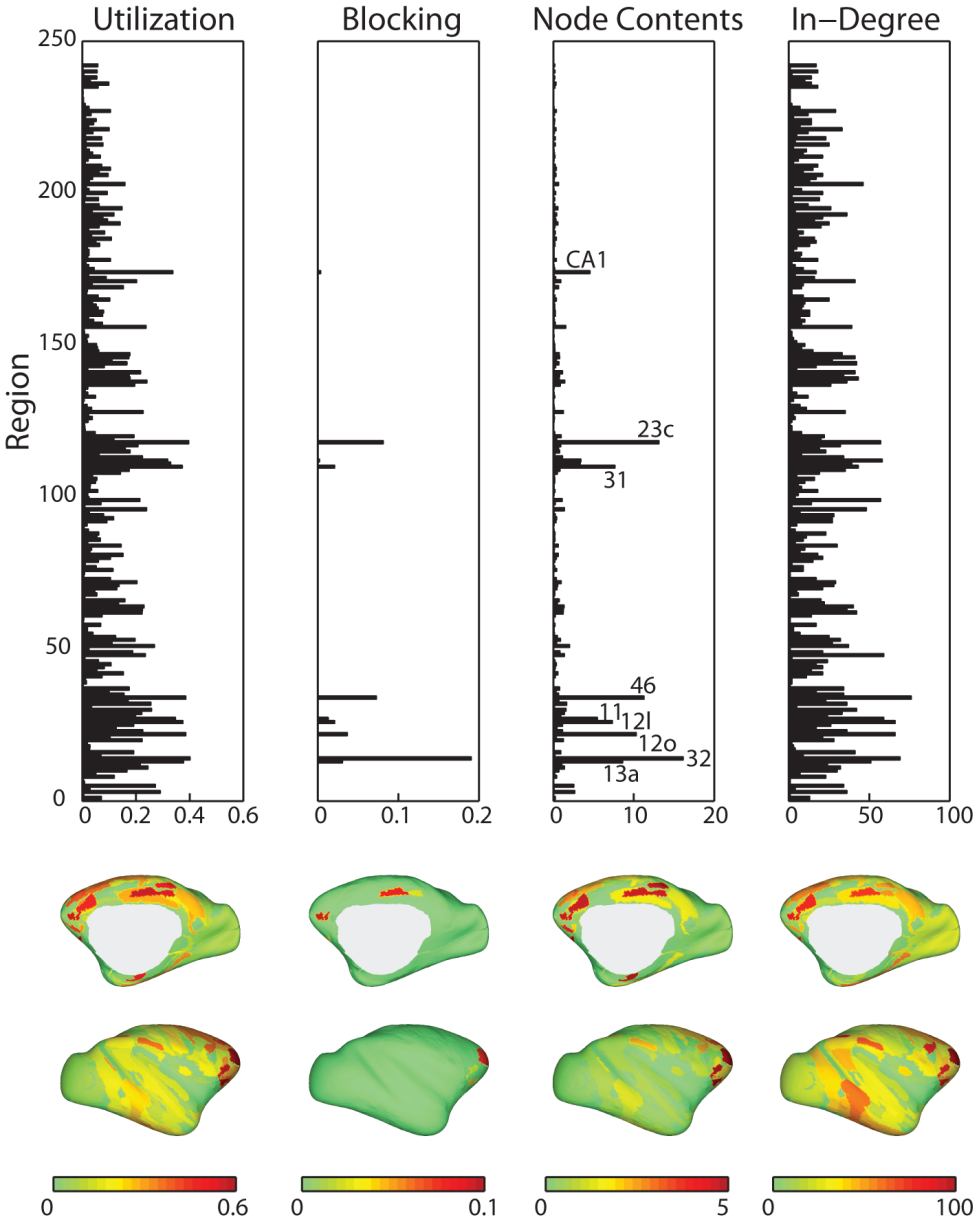
Node-level statistics. Top: Utilization, blocking probability, node contents and in-degree are shown for each of the 242 nodes on the CoCoMac network, averaged over 500 simulations (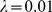, 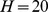, 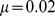). For illustrative purposes, nine nodes with the highest node contents are labeled. Bottom: Inflated surface renderings showing the anatomical distribution of each statistic, for the medial and lateral surfaces.

We now examine regional contributions of the CoCoMac network in detail. To study the individual relevance of nodes for information flow, three complementary node-level metrics of congestion were used: utilization, blocking and mean node contents. Information flow was highly heterogeneous across the network, with some nodes vulnerable to overwhelming influx, while others experienced only occasional traffic (Fig. 3). To a large extent congestion at a given node was predicted by the number of afferent projections to that node (in-degree, 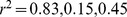 and 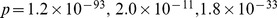 for utilization, blocking and node contents, respectively) and this is expected given the fact that in the present model information flow is implemented as an interactive random walk [2], [20], [21]. With the exception of CA1, all nodes with the largest average contents were previously identified as part of the rich club [17](Fig. S2), indicating that membership in this densely inter-connected subgraph entails a heavy workload. Much of the congestion appears to be concentrated at three distinct sites, mainly along the medial surface, roughly corresponding to medial prefrontal cortex, medial/inferior temporal cortex and precuneus/posterior cingulate cortex.

**Figure 4:**
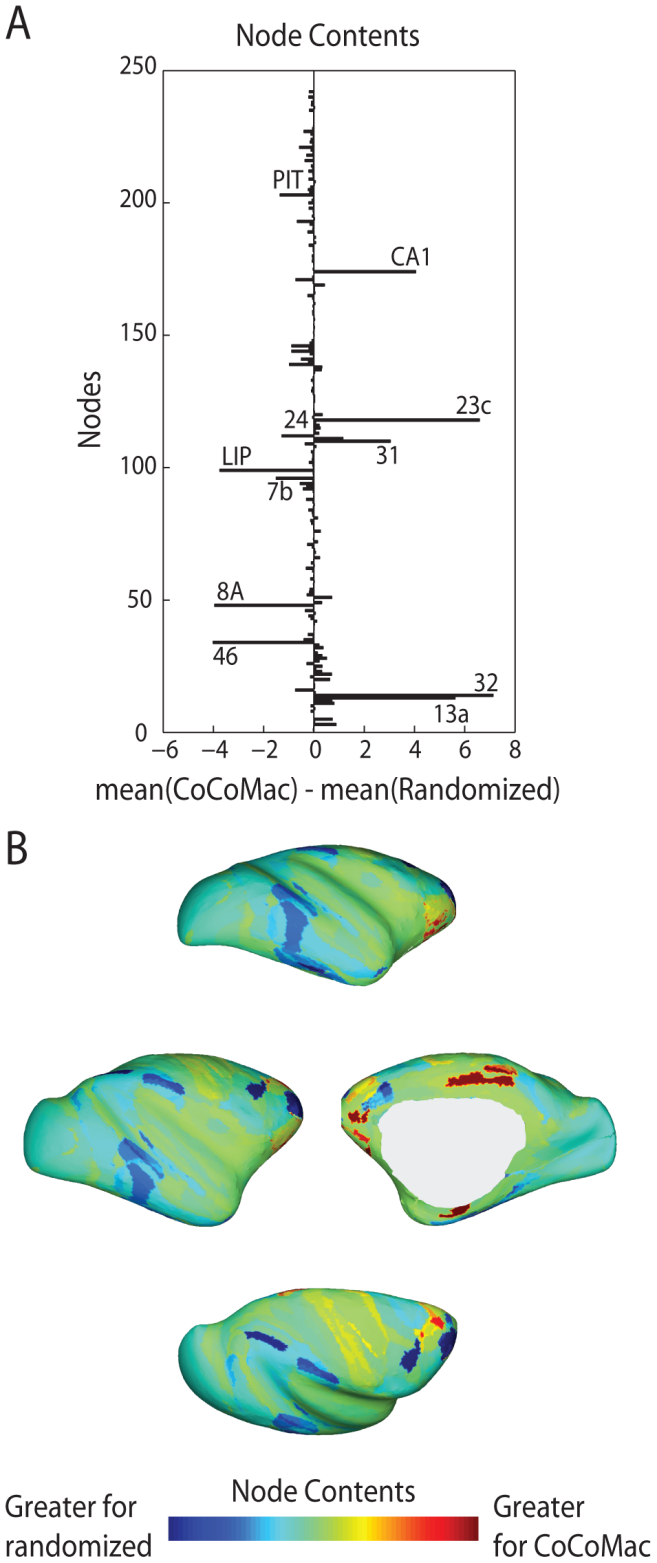
Node contents for the CoCoMac and randomized control networks. (A) The mean differences in node contents between 100 simulations based on the CoCoMac network, and 100 simulations on 100 randomized networks (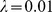, 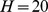, 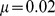). Nodes with statistically significant differences are labeled. (B) Surface renderings of the mean differences in node contents.

To determine the extent to which these congestion metrics depend on topology rather than degree sequence, we statistically compared them to a set of metrics from simulations run on a population of randomized control networks for which the topology had been altered while preserving degree sequence [22]. Fig. 4A shows the “raw” mean differences between the two networks for the contents of each node, while Fig. 4B shows the spatial distribution of these differences. Due to the high level of consistency between the three metrics of congestion (utilization, blocking and contents), only the results for node contents are shown. Nodes with contents that are significantly different (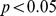, controlled for multiple comparisons using false-discovery rate correction) for the two sets of the networks are labeled. Interestingly, while the majority of these nodes are part of the rich club, nearly half experience greater congestion in the macaque network, while half experience greater congestion in the randomized networks. This suggests that macaque cortical connectivity imposes a characteristic set of traffic patterns, such that signal traffic is directed towards some nodes and away from others, in contrast to what would be expected based only on the degree of these nodes.

### Edge statistics

**Figure 5:**
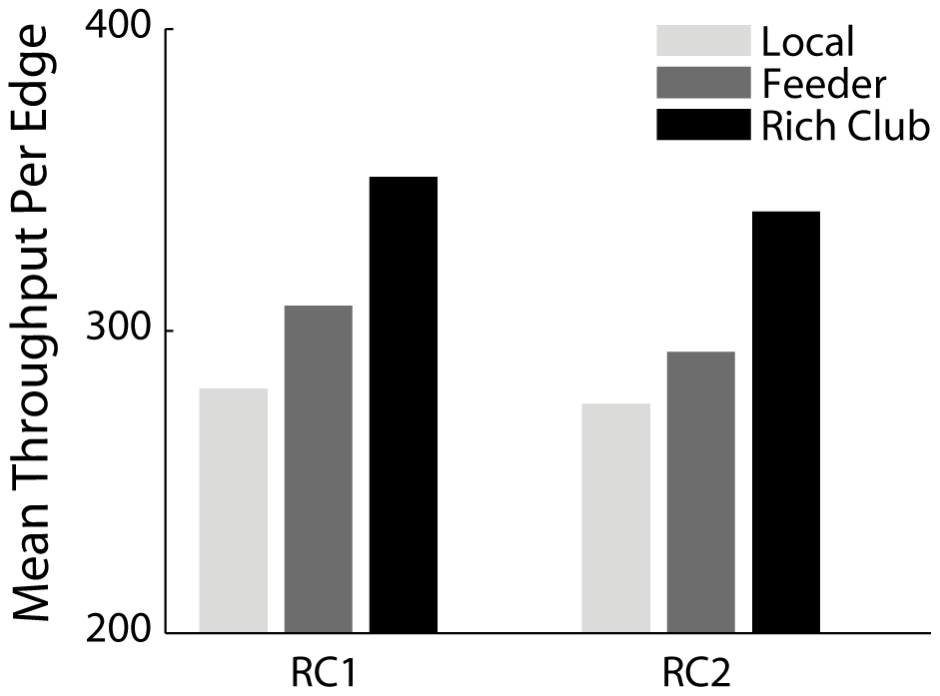
Edge throughput. The mean number of signal units carried by each edge. Edges are classified as Rich Club (R), Feeder (F) or Local (L). Results are shown for 500 simulations (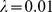, 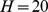, 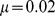) on the CoCoMac network.

We next consider information flow with respect to specific edges. Given the relevance of the rich club in the macaque network [17], we classified edges according to whether they connect rich club nodes [16]. Edges connecting two non-rich club nodes were classified as (L)ocal, those connecting a non-rich club and a rich club node as (F)eeder and those connecting two rich club nodes as (R)ich club. Moreover, these classifications were made with respect to two rich club levels, RC1 and RC2, which represent a more conservative and a more liberal definition of the rich club [17]. An initial observation is that this stratification of edges closely resembles the patterns of edge throughputs. In particular, projections with greater throughput appear more likely to be those connected to at least one rich club node, i.e. Rich Club or Feeder. Despite the fact that the vast majority of edges in the macaque network are Local, followed by Feeder and then Rich Club [17], the mean throughput per edge is greatest for Rich Club edges, followed by Feeder and Local (Fig. 5B). In other words, traffic tends to concentrate not just at rich club nodes, but also at the edges around them, effectively encompassing their local neighborhoods.

### Path statistics

**Figure 6:**
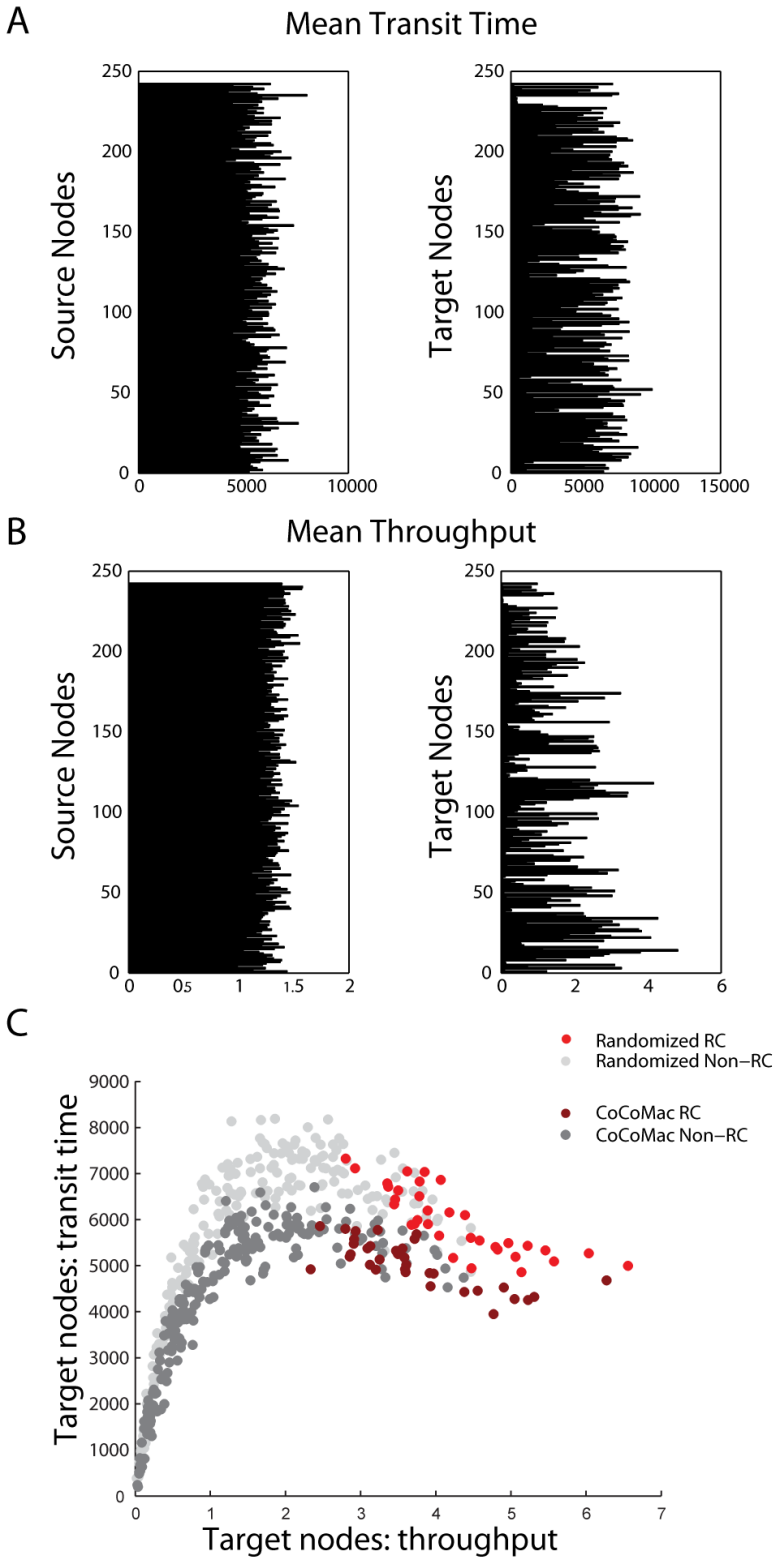
Delays and deliveries. Results are shown for 500 simulations on the CoCoMac network (A,B,C) and 1 simulation on 500 randomized networks (C) (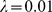, 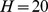, 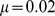). Means of transit times (A) and throughput (B) are either taken across all target nodes (showing the mean for individual source nodes), or across source nodes (showing the mean for individual target nodes). (C) The relationship between transit time and throughput across all target nodes.

Finally, we investigate information flow with respect to every possible pair of source and target nodes. For each pair, all completed trajectories are compiled in order to compute the total number of deliveries (throughput), as well as their mean transit time or delay. For both the throughput and transit time statistics, taking the mean across sources results in greater variance than taking the mean across targets (Fig. 6A,B). For the target nodes, both statistics showed substantial association with in-degree (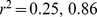, 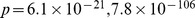 for transit time and throughput, respectively). In other words, the mean throughput and transit time were much more dependent on the destination, rather than the source, indicating that some nodes in the network are intrinsically easy to reach, while others are intrinsically difficult.

A non-monotonic relationship emerges when comparing the mean throughput and the mean transit time across target nodes (Fig. 6C, dark grey). When the total throughput is low, any increase in throughput results in slower transit times. However, for a subset of nodes with a high throughput this relationship does not hold and these nodes tend to receive information much faster than would be expected. Most of these nodes belong to the rich club (RC2), indicating that rich club nodes receive more information than other nodes in the network, and do so with a disproportionately faster latency. A similar relationship is observed for degree-preserving randomized controls (Fig. 6C, light grey), indicating that the effect is largely due to the high degrees of rich club nodes. Rich club connectivity enhanced the effect. As expected from Fig. 2, the randomized controls generally have slightly higher throughput, but also longer transit times. This was particularly true for rich club nodes, which received significantly fewer signal units when embedded in the macaque than in randomized networks (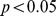 for RC1 and RC2), but did so with significantly faster transit times (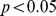 for RC1 and RC2).

## Discussion

The complex anatomical connectivity of the central nervous system suggests that inter-regional communication is important for the functioning of the brain, and in the present report we systematically investigated the effect of network topology on communication. Utilizing a modeling paradigm from telecommunications and statistical physics [2], [21], we superimposed a communication system on an empirically derived network describing macaque cerebral cortex. Our results highlight multiple ways in which structural connectivity has the potential to exert considerable influence on information flow in brain networks.

In terms of global information flow statistics, the macaque network was found to be intermediate to its latticized and randomized reference networks, mirroring the notion that the complex topology of structural networks represents a trade-off between wiring cost and communication efficiency for integrative processing [11], [12], [23]. In particular, the macaque network exhibited an economic balance between speed, fidelity, utilization and sheer volume of transmission. Compared to degree-matched random networks, the macaque network appeared to prioritize speed of transmission over throughput and reliability.

Although many studies have reported evidence of small world organization in structural [6]–[8] and functional networks [24], [25], a canonical small world model by itself could not account for the information processing characteristics observed in the macaque network. However, the added presence of a rich club largely replicated the information flow signature of the macaque network. Therefore, our data suggest that the small world property (in the Watts-Strogatz sense [19]), together with the rich club, is necessary to produce the macaque-like pattern, but by itself is not sufficient. Several recent studies have postulated that a densely interconnected rich club has the potential to facilitate global integration by providing an easily accessible high-capacity backbone that serves to attract and disseminate interregional signal traffic [14]–[17], [26]. By demonstrating that the rich club is a principal topological feature with respect to communication dynamics, our model lends further support to this notion.

Forming a triangle spanning frontal cortex, posterior cingulate cortex/precuneus and medial temporal cortex, the rich club subgraph proved to be a prominent axis in the information processing architecture of the network. Rich club nodes, as well as connections involving rich club nodes, absorbed the greatest signal traffic, indicating that the rich club of densely connected hubs supports the capacity to efficiently centralize and, presumably, integrate information. The fact that rich club nodes are more likely to exhibit signs of congestion warrants further investigation into their potential role as bottlenecks in information processing. Several empirical studies have reported evidence of bottlenecks limiting processing capacity in attention [18] and response selection [27], and the areas they implicate show considerable correspondence with rich club regions (including medial prefrontal cortex and precuneus), although a direct comparison between the macaque network and human fMRI studies is difficult.

Despite the fact that rich club nodes were among the most congested, comparisons with surrogate networks revealed that rich club connectivity may serve to shape information flow, whereby signal traffic is biased towards some nodes and away from others. While a number of rich club nodes consistently experienced heavier traffic than would be expected on the basis of their degrees, others consistently experienced lighter traffic than would be expected on the basis of theirs. Why macaque network topology, and the rich club in particular, shapes cortico-cortical communication in a way that imposes this specific pattern of information flow, remains unclear. It is noteworthy that the under-congested nodes are areas associated with making eye movements, tracking and acting towards objects in space and fusing visual and proprioceptive information. Many of these areas are part of the dorsal attention sub-network [28]–[30], which presumably must be continually responsive and capable of rapidly integrating and communicating information. We therefore speculate that the topology of the global network is configured in a way that relieves congestion at these dorsal attention areas to facilitate fast and efficient interaction with the external environment.

Previous analyses have shown that, despite constituting only a small part of total network density, rich club connections participate in the greatest number of shortest paths in the network [16], [17]. This has led to the hypothesis that if rich club topology is configured in a way that facilitates cortico-cortical communication via shortest paths, there may exist a set of routing or navigation strategies to take advantage of this feature [16], such as “greedy” routing [31]. However, our data demonstrate that the rich club is central to global communication even if information flow is governed by simple diffusion rather than shortest path communication, potentially eschewing the need for a more complex routing mechanism. To characterize the organizational principles of global information flow, further investigation is necessary to determine which types of routing strategies can best take advantage of the unique connectivity of brain networks, as well as which types of routing strategies best replicate empirical functional data.

### Methodological limitations

The modeling paradigm employed in the present study entails a number of features and simplifying assumptions. Therefore, it is important to consider what the biological correlates of these features are and the extent to which they limit the utility of the model. Central to our approach are discrete signal units. At the large spatial scale, it is unlikely that neural communication takes place via discrete signal units. Rather, information flow between large scale neuronal ensembles is likely to be based on spike trains or coordinated volleys of spike trains. It is also possible that information is transferred as an ensemble of signals from multiple neurons. In our model signal units represent the ability of brain regions to influence one another. This simplifying assumption allows us to trace the trajectory of each signal unit as it propagates in the network, and hence to calculate various metrics about the potential for communication that is afforded by the anatomical connectivity.

External arrivals represent the assumption that new information is continuously generated and communicated in the network. The source of this information may be either stimulation exogenous to the nervous system, or some endogenous process. Poisson arrivals were chosen because at the level of individual neurons, inter-spike intervals (ISIs) are found to be exponentially distributed [32] and likewise, in psychophysics and signal detection, the Poisson process is often used to model stimulus fluctuations and other statistical properties of the sensory environment [33]. Queues and finite buffers are constructs that allow us to model how network topology constrains information flow. Queueing is a mechanism by which signal units are made to interact as they flow through the network, modeling the interplay between multiple information flows on top of the structural network [34]. Finite buffers allow for the possibility of signal loss, modeling the poor fidelity of neural transmission [35].

Note also that the present model does not take into account the intrinsic hierarchy of sub-domains of brain networks. For instance, one may expect primary sensory areas to send more information than they receive. Likewise, one may expect higher order, multimodal areas to receive more information than they send. In our model, source and destination nodes for each signal unit are chosen randomly, irrespective of their function, and we largely ignore this aspect of cortical organization. Future studies should investigate this fundamental feature of cortical networks.

The strength of the modeling approach pursued here is that it allows one to generate relative metrics about network communication. The approach is complementary to other, more physiologically realistic paradigms for modeling global system dynamics [36]–[41], which do not model information transmission directly. These models suggest that three key ingredients are needed to generate realistic brain dynamics: empirically derived patterns of structural connectivity, time-delayed transmission and noise [40], [41]. Indeed, a queueing network model has the potential to incorporate all three, and the present implementation includes both empirically derived connectivity and stochastic dynamics.

A fundamental aspect of networked communication is the switching architecture: the manner in which information is directed and transported across the network. In the present study, we utilized a message-switched architecture, wherein an entire “message” is contained in a single discrete signal unit. Our study represents the first attempt to characterize communication dynamics in brain networks, so this type of switching architecture, together with diffusive, stochastic routing, was particularly advantageous because this type of model does not assume that signal units have any knowledge of the global topology or traffic conditions [42]–[44]. A physiologically plausible alternative would be a packet-switched architecture, wherein a message is broken up into packets, which individually take the most efficient path to the destination, where they are re-assembled [45]. This type of architecture has many potential advantages for systems that rely on temporally sparse “bursts” of communication, including lower transit times [45]. Thus, our results and conclusions are strongly tied to the diffusion-based, non-hierarchical, message-switched architecture we used, and may not hold for other switching architectures.

### Conclusion

Altogether, our results reveal a dynamic aspect of the global information processing architecture and the critical role played by the so-called “rich club” of hub nodes. Our work lays the foundation for further systematic study of organizational principles for communication in large-scale brain networks, including routing strategies and resource allocation.

## Materials and Methods

### Anatomical and reference networks

The anatomical connectivity data set used in the present study was derived from the online Collation of Connectivity data on the Macaque brain (CoCoMac) database, comprised of data from 413 tract tracing studies of the macaque [46], [47]. The database was originally queried by [48] and further condensed by [17]. To facilitate comparison with previous reports, only cortical nodes were included. The final directed network was comprised of 242 nodes and 4090 edges and was fully connected, such that each node maintained at least one incoming and one outgoing edge.

Two populations of surrogate networks - one randomized and one latticized - were generated to explore the extent to which the topology of the macaque connectivity matrix influenced the simulation results. Randomized networks were generated using a Markov switching algorithm that randomly swapped pairs of edges [22]. Latticized networks were generated using a modified version of the same algorithm, whereby the edges were swapped only if they moved closer to the main diagonal as a result [7]. By randomly re-ordering edges and forcing them closer to the diagonal, the topology of the original network is destroyed and replaced by one where neighbouring nodes are more likely to be connected, as in a ring lattice. Both sets of surrogate networks were degree-matched in the sense that the in-degree and out-degree of each node was preserved. Statistical assessment was performed by comparing 100 simulations on the CoCoMac network with 100 simulations on a randomized null network, for 100 null network realizations. Comparisons between node-specific metrics were made using Welch's *t*-test for samples with unequal variances [49], and evaluated with respect to degrees of freedom determined using the Satterthwaite approximation [50]. To control the false discovery rate, 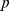-values were corrected following the procedure outlined by [51].

A similar procedure was performed for synthetic small-world [19] and rich club [14], [15], [52] networks and their respective null models. A network containing a rich-club was created from a random network by endowing a sub-set of the nodes (the rich club) with greater connection density than the rest of the network, and an even greater connection density amongst each other. The randomized and latticized controls were then created as described above. For the small world scenario, the starting point was a ring lattice. A small world network was generated by randomly permuting 10% of the edges, while a completely randomized network was generated by further permuting each edge 100 times.

### Rich club detection

Our results have considerable implication for the rich club feature of brain network topology and so for completeness we briefly rehearse the procedure for detecting and defining rich clubs. Fuller descriptions of the rich club phenomenon can be found elsewhere, for brain networks in general [15], [16], as well as for this particular network [17].

For a given graph, a rich club is defined as a set of high-degree nodes (a subgraph) that are more densely connected amongst each other than would be expected on the basis of degree alone [52]. Rich club classification is made with respect to a range of node degrees. For a given degree 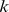, all nodes with degree 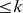 are stripped from the network. A rich club coefficient 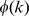 is calculated as the ratio of remaining connections to all possible connections. Thus, 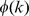 can be thought of as the density of the subgraph. For the same set of nodes, the ratio is also computed with respect to 10,000 degree-matched randomized networks. The normalized rich club coefficient, 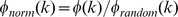, measures the density of the subgraph relative to the null model where the global topology has been destroyed. These steps are repeated for a range of 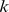, from the lowest to the second-highest degree in the macaque network (2 to 121). A 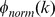 consistently greater than 1 for a range of 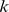 suggests the existence of rich club organization.

Therefore, across the range of 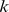 it is possible to define unique sets of rich club nodes corresponding to different values of 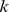. These nodes can then be positioned in a nested hierarchy of rich club “levels”, ranging from those containing nodes with the highest degree to those containing nodes with the lowest degree. In the present study, we follow the classification made by [17], whereby two rich clubs were singled out. The first, RC1, was more densely interconnected and comprised of fewer nodes, with greater minimum degree. The second, RC2, was less densely interconnected and contained more nodes, with smaller minimum degree. RC2 is a subset of RC1, and by examining these two levels of the rich club, it is possible to identify robust relationships between rich-club organization and information flow as estimated by our model.

Once nodes have been classified as either rich club or non-rich club, it becomes possible to classify edges as well. Namely, edges that connect non-rich club nodes to non-rich club nodes are classified as “local”, those connecting non-rich club nodes to rich club nodes as “feeder” and those connecting rich club nodes to other rich club nodes as “rich club”.

### Discrete-event simulation

Signal units were generated and introduced in the network according to a Poisson process with rate 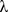, i.e. with exponentially distributed inter-arrival times. For each signal unit, a source node and destination node were randomly selected. To reach its destination node, the signal propagated to one of the neighbouring nodes, with equal probability for each. The time spent at each node (service time) was exponentially distributed with rate 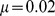. If a signal unit arrived at a node that was occupied, a queue was formed. Units entered the node on a last-come-first-served basis, also known as last-in-first-out (LIFO) queueing [53]–[55]. A maximum buffer size was imposed (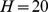), such that a signal unit arriving at a full buffer caused the oldest signal unit in the queue to be ejected and removed from the system. Upon reaching the destination node, the unit was removed from the network. The purpose of queueing is simply to ensure that information flow is interactive, while a finite buffer size allowed us to model imperfect signal transmission [35]. Buffer capacity is not a critical parameter, in the sense that it cannot induce a phase transition in the system. Changes in buffer capacity will produce quantitative, but not qualitative, changes in system behavior (SI Section 3, Fig. S5).

This type of model has two characteristic modes of operation. At low intensities (external arrival rates), the total number of signal units in the network fluctuates around some finite value and the system is said to be in a steady-state. As the intensity is increased, there is a qualitative change in the system dynamics, characterized by a monotonic increase in the number of signal units in the network until all buffers are filled, leading to “jamming” [2], [20]. The key variable is the ratio between the arrival rate and service rate at each node. Therefore, we fixed the service rate (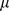) and varied the rate of external arrivals (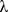). The focus of the present study was on the steady-state behavior of the network, and the range of external arrival rates (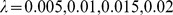) was chosen to sustain stationary flow, prior to the phase transition.

All simulations were run for 2 million dimensionless time units. Due to the presence of stochastic time variables in the simulation (inter-arrival times and service times), the state of the system was updated at non-uniform time points. Upon completion, the time series of system states were linearly interpolated to produce uniformly sampled time series (Text S1, Section 6, Fig. S6). An initial transient of 40,000 time units, during which the system state had not yet stabilized (determined via the ensemble average method [54]), was discarded from further analysis to avoid transitory effects. The Mersenne Twister [56] was used to generate a uniform distribution, which was then used to generate exponentially distributed random numbers (inter-arrival times and service times) using the standard inverse transform method. All simulations were implemented in Matlab (Mathworks Inc., Natick, MA) and independently verified in Artifex (RSoft Design Group Inc., Ossining, NY), as well as analytically (Text S1 Section 2, Figs. S3,4).

All signal units were uniquely identified, allowing for their position and complete trajectory in the network to be traced across the simulation. These trajectories were then analyzed to compile a set of node-, edge- and network-level statistics. For each node, we calculated the mean proportion of time the node was busy (utilization), the probability of signal loss (blocking) and the mean system contents. For each edge, we calculated the mean throughput of signal units. For each network, we calculated the mean utilization and blocking rates across nodes, as well as the total number of signal units successfully transmitted from source to destination (throughput) and the mean latency of those transmissions (transit time).

More formally, simulation variables were defined as follows. A node 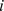 at time 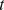 has two components: the server contents 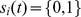, which describes the number of signal units currently in service, and the queue length 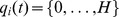, which describes the number of signal units waiting in the buffer. The node contents 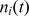 were thus defined as
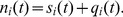
(1)

Likewise, the contents at any existing channel from node 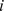 to node 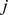 was 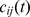. The total network load 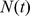 is then the sum of all node and channel contents:
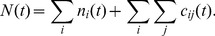
(2)

The utilization of node 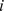 is the proportion of simulation time during which 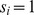. The blocking probability at node 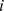 was calculated as the number of signal units ejected from 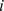 divided by the total number of signal units arriving at 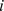.

The total time a signal unit spends at a single node, 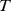, is the sum of the waiting time in the queue 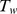 and the service time in the node 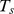

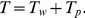
(3)

Both 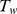 and 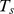 are stochastic processes, with 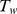 determined by the the topology and dynamics on the network, while 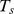 is drawn from an exponential distribution with rate 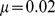. For any signal unit, the transit time is the sum of waiting and service times across all nodes traversed from source to destination. Transit time statistics are calculated only for signals that successfully reached their destination.

## Supporting Information

Figure S1**Effect of increasing simulation intensity.** Fluctuations in the total number of signal units present in the network during a single simulation run, shown for three different arrival rates (simulation intensities, 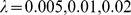).(TIF)

Figure S2 **Rich club of the macaque network.** The spatial distribution of the rich club, shown for two different rich club “levels” (adapted from [17]).
(TIF)

Figure S3**System statistics: analytical and numerical results.** The network-level results of the analytical model are shown against the numerical simulation, with 500 replications (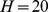, 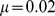).(TIF)

Figure S4**Node statistics: analytical and numerical results.** The node-level results of the analytical model are shown against the numerical simulation, with 500 simulations (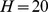, 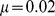).(TIF)

Figure S5**Effect of buffer size.** The results of 500 simulations (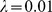, 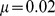), showing the utilization, blocking and node contents at each node for three different buffer sizes: 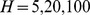.(TIF)

Figure S6**Effect of interpolation.** Network load time series are shown for a single simulation (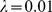, 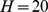, 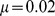). (A) Original time series, with non-uniform sampling. (B) Linearly interpolated time series, with uniform sampling. (C) Interpolated time series overlayed on the original time series.(TIF)

Figure S7**Effect of rich club size.** System statistics for 500 simulations (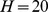, 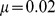) for three different synthetic “rich club” networks, with rich clubs comprised of 10, 20 and 30 nodes, out of 100 total nodes.(TIF)

Table S1**Comparing scenarios.** To assess similarities in the patterns of results produced by the CoCoMac, Small-World and Rich-Club scenarios, we correlate the values of specific network metrics (transit time, throughput, utilization and blocking) across networks (original, randomized and latticized) and simulation intensities (0.05, 0.10, 0.15 and 0.20). Fisher's 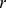-to-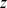 expresses the difference between the CoCoMac-Small World and CoCoMac-Rich Club correlation coefficients as a 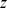-score. Values greater than 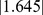 indicate that the CoCoMac-Rich Club correlation is significantly greater than the CoCoMac-Small World correlation.(PDF)

Table S2**Network comparisons for the transit time statistic.** The average of 100 simulations on the CoCoMac (C) network was compared against 100 simulations on randomized (R) and latticized (L) null networks, for 100 null network realizations. The entries represent the average 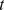-statistics and 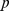-values for those 100 comparisons.(PDF)

Table S3**Network comparisons for the throughput statistic.** The average of 100 simulations on the CoCoMac (C) network was compared against 100 simulations on randomized (R) and latticized (L) null networks, for 100 null network realizations. The entries represent the average 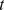-statistics and 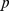-values for those 100 comparisons.(PDF)

Table S4**Comparisons for the node contents statistic.** The average of 100 simulations on the CoCoMac network was compared against 100 simulations on randomized networks, for 100 null network realizations. The entries represent nodes with statistically significant differences, and the average 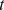-statistics and 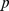-values for those 100 comparisons.(PDF)

Text S1 **Supporting information text.** The supporting information contains the following: explanation of the biological meaning of various model components (Section 1), an analytical model (Section 2), exploration of parameter space (Section 3), a statistical assessment of the similarity between network scenarios (Section 4), exact T- and P-values (Section 5), assessment of the effect of resampling simulation time series (Section 6) and an assessment of the effect of rich club size (Section 7).
(PDF)
