# Supplementary figures and images for: Communication Efficiency and Congestion of Signal Traffic in Large-Scale Brain Networks
Source: PLoS Comput Biol. 2014 Jan 9;10(1):e1003427. doi: 10.1371/journal.pcbi.1003427 (PMC3886893; doi:10.1371/journal.pcbi.1003427)

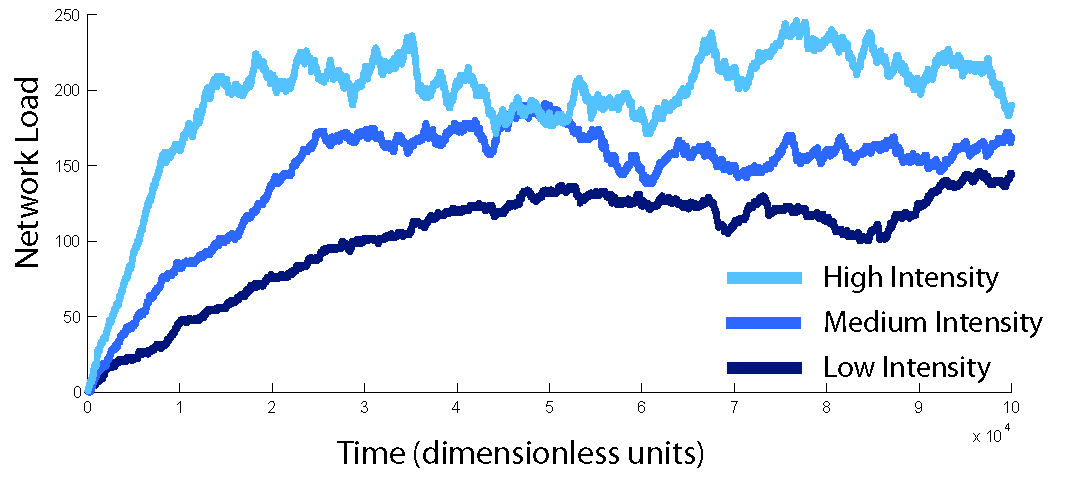

Supplement: Figure S1 — Effect of increasing simulation intensity. Fluctuations in the total number of signal units present in the network during a single simulation run, shown for three different arrival rates (simulation intensities, ). (TIF) [file pcbi.1003427.s001.tif]

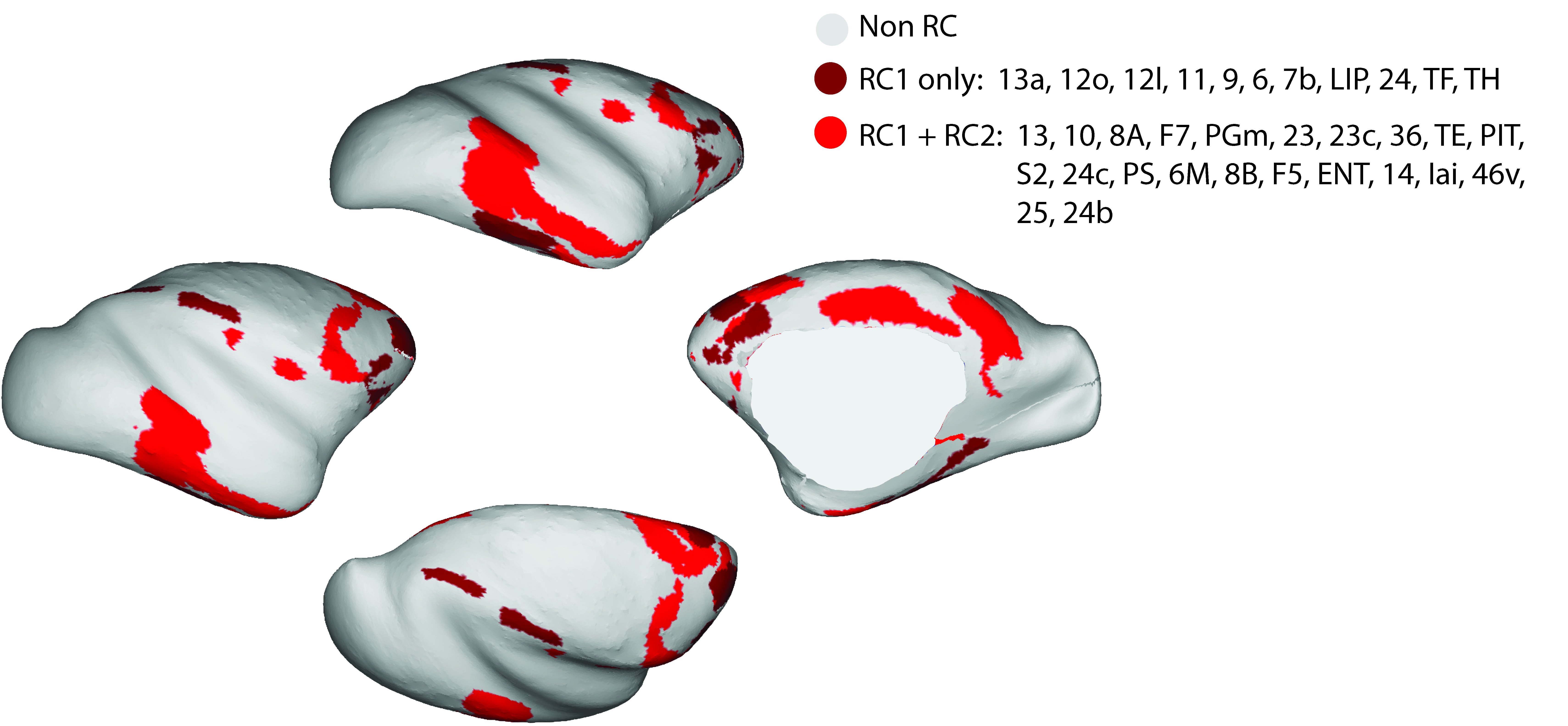

Supplement: Figure S2 — Rich club of the macaque network. The spatial distribution of the rich club, shown for two different rich club “levels” (adapted from [17]). (TIF) [file pcbi.1003427.s002.tif]

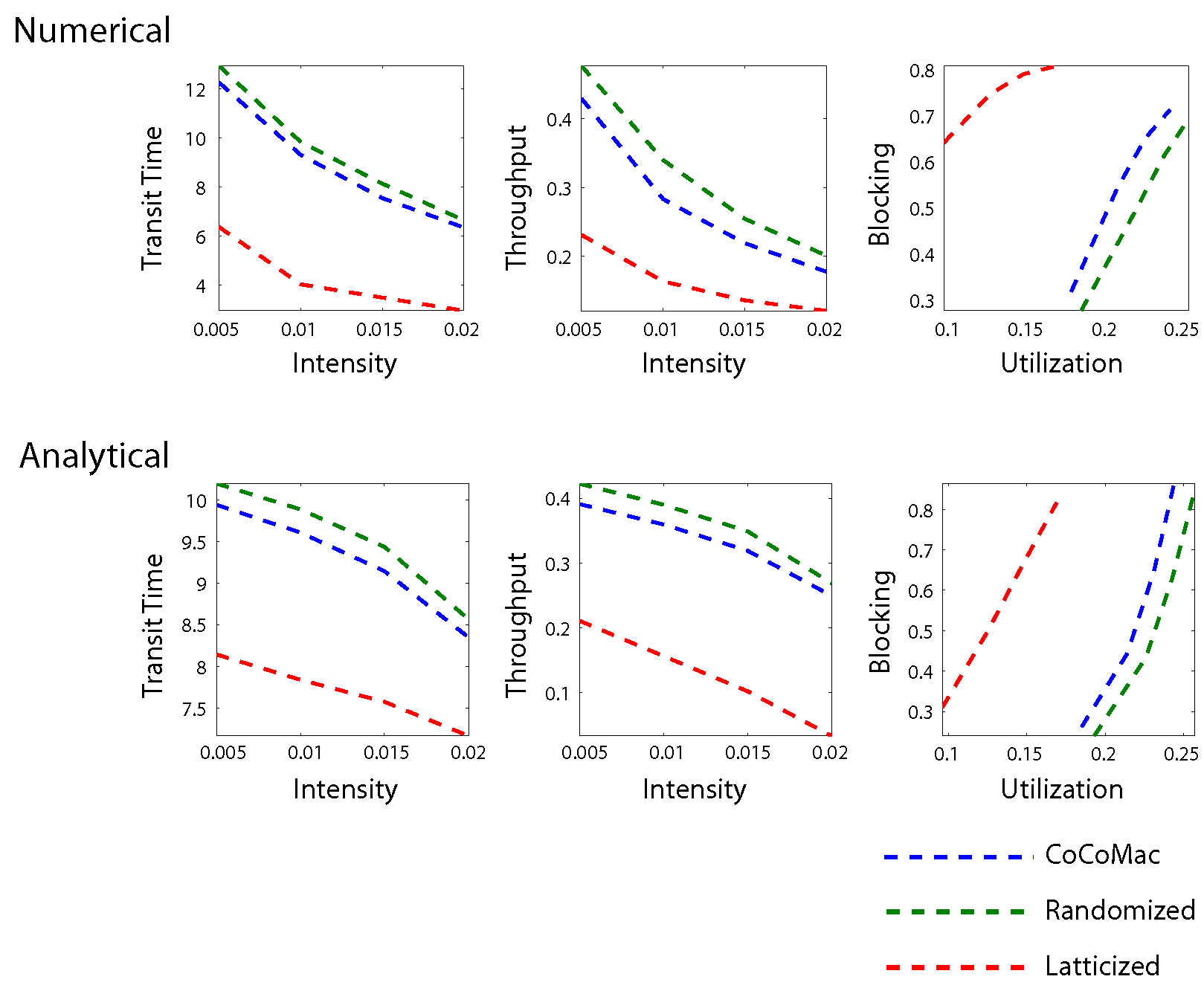

Supplement: Figure S3 — System statistics: analytical and numerical results. The network-level results of the analytical model are shown against the numerical simulation, with 500 replications (, ). (TIF) [file pcbi.1003427.s003.tif]

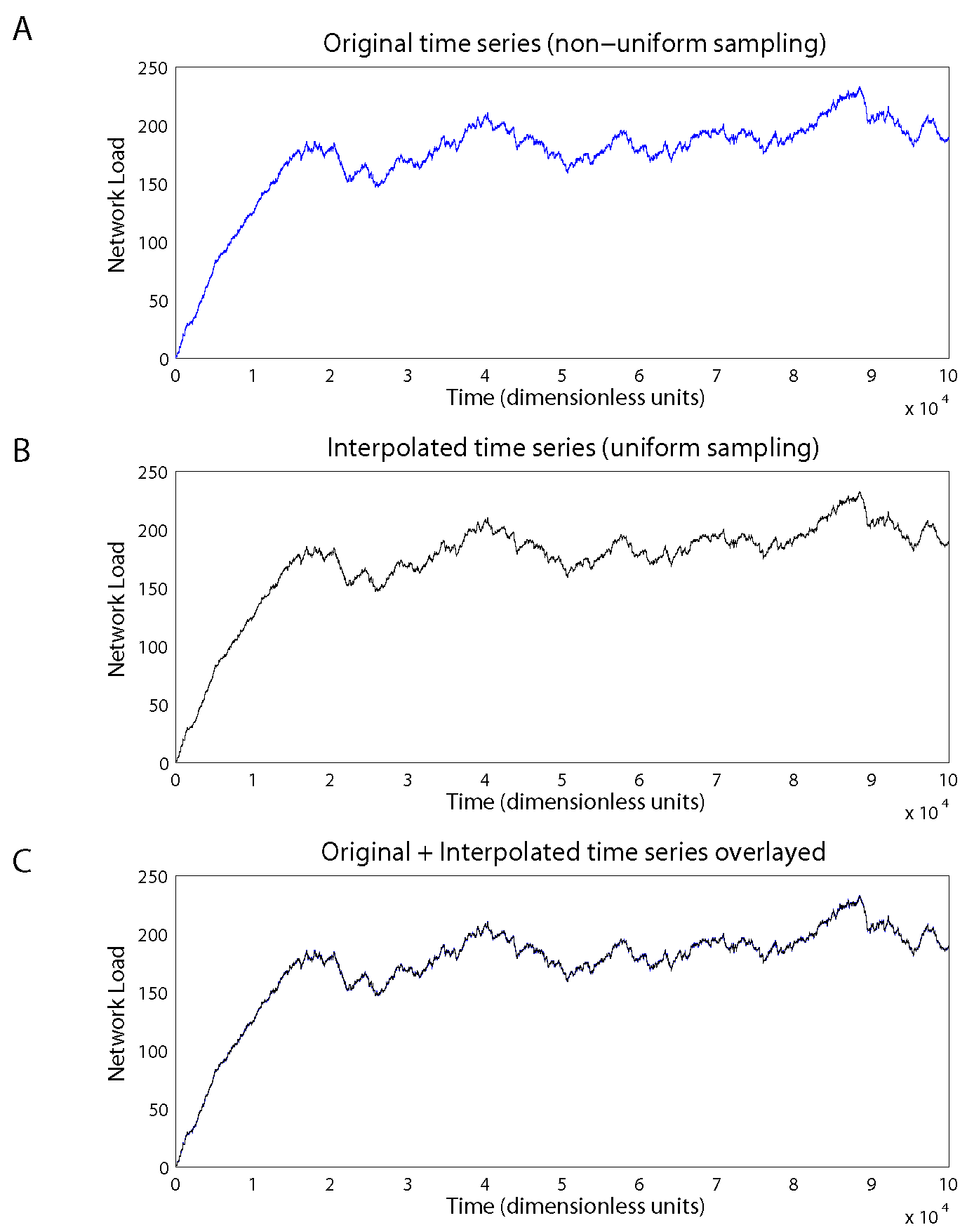

Supplement: Figure S6 — Effect of interpolation. Network load time series are shown for a single simulation (, , ). (A) Original time series, with non-uniform sampling. (B) Linearly interpolated time series, with uniform sampling. (C) Interpolated time series overlayed on the original time series. (TIF) [file pcbi.1003427.s006.tif]

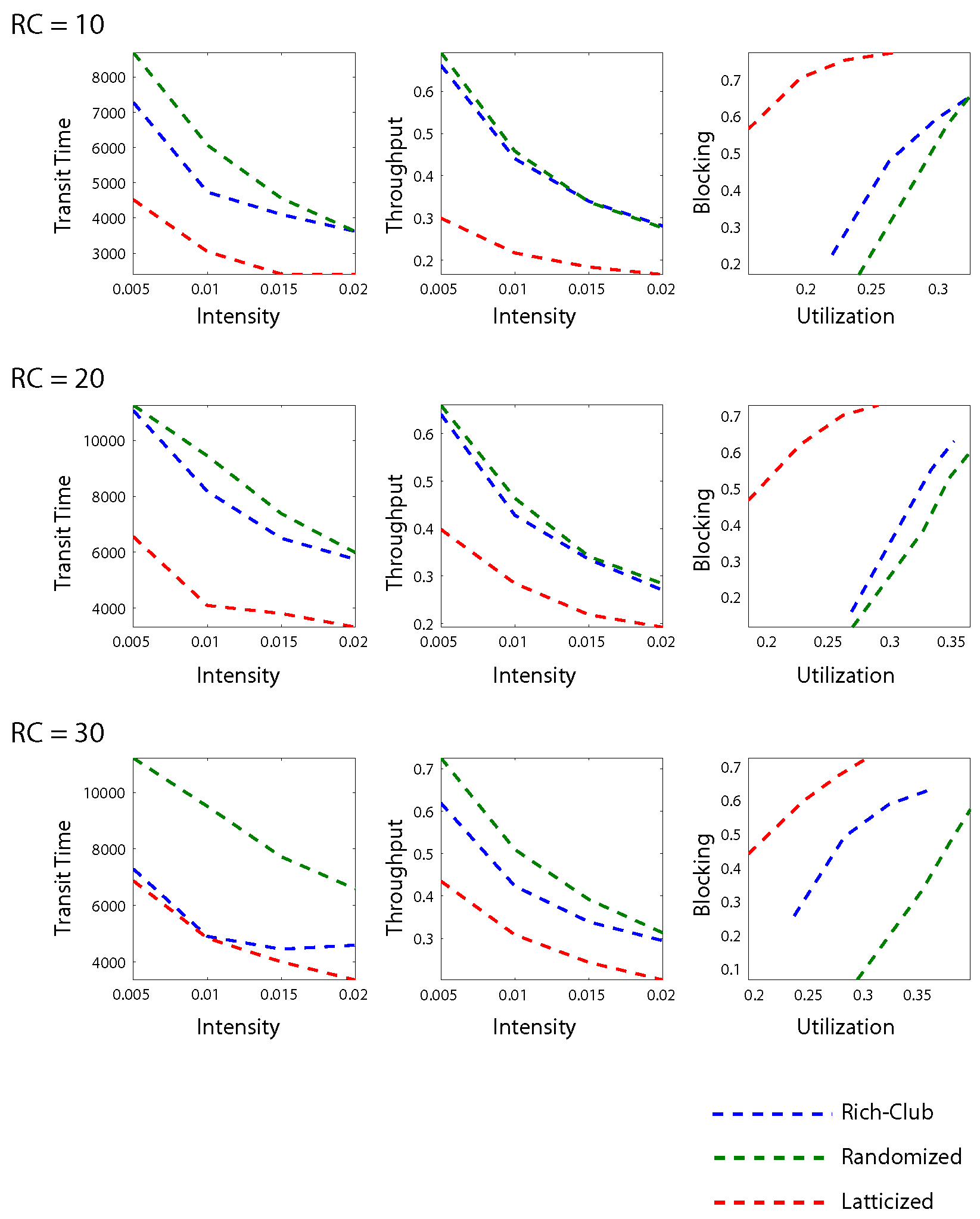

Supplement: Figure S7 — Effect of rich club size. System statistics for 500 simulations (, ) for three different synthetic “rich club” networks, with rich clubs comprised of 10, 20 and 30 nodes, out of 100 total nodes. (TIF) [file pcbi.1003427.s007.tif]
