# Supplementary material for: Communication Efficiency and Congestion of Signal Traffic in Large-Scale Brain Networks
Source: PLoS Comput Biol. 2014 Jan 9;10(1):e1003427. doi: 10.1371/journal.pcbi.1003427 (PMC3886893; doi:10.1371/journal.pcbi.1003427)
Supplement: Table S1 — Comparing scenarios. To assess similarities in the patterns of results produced by the CoCoMac, Small-World and Rich-Club scenarios, we correlate the values of specific network metrics (transit time, throughput, utilization and blocking) across networks (original, randomized and latticized) and simulation intensities (0.05, 0.10, 0.15 and 0.20). Fisher's -to- expresses the difference between the CoCoMac-Small World and CoCoMac-Rich Club correlation coefficients as a -score. Values greater than indicate that the CoCoMac-Rich Club correlation is significantly greater than the CoCoMac-Small World correlation. (PDF) [file pcbi.1003427.s008.pdf]

	Transit Time	Throughput	Utilization	Blocking
CoCoMac vs	$r = 0.78$	$r = 0.88$	$r = 0.88$	$r = 0.86$
Small-World	$p < 0.01$	$p < 0.001$	$p < 0.001$	$p < 0.001$
CoCoMac vs	$r = 0.99$	$r = 0.98$	$r = 0.98$	$r = 0.99$
Rich-Club	$p << 0.001$	$p << 0.001$	$p << 0.001$	$p << 0.001$
Fisher's r-to-z	$z = 3.08$	$z = 2.14$	$z = 3.67$	$z = 2.27$
