# Supplementary material for: Communication Efficiency and Congestion of Signal Traffic in Large-Scale Brain Networks
Source: PLoS Comput Biol. 2014 Jan 9;10(1):e1003427. doi: 10.1371/journal.pcbi.1003427 (PMC3886893; doi:10.1371/journal.pcbi.1003427)
Supplement: Table S3 — Network comparisons for the throughput statistic. The average of 100 simulations on the CoCoMac (C) network was compared against 100 simulations on randomized (R) and latticized (L) null networks, for 100 null network realizations. The entries represent the average -statistics and -values for those 100 comparisons. (PDF) [file pcbi.1003427.s010.pdf]

intensity	C vs R	C vs L	R vs L
0.005	$t = -2.81$<br>$p = 6.0 \times 10^{-3}$	$t = -12.95$<br>$p = 5.1 \times 10^{-23}$	$t = -11.63$<br>$p = 3.2 \times 10^{-20}$
0.010	$t = -3.07$<br>$p = 2.7 \times 10^{-3}$	$t = -15.28$<br>$p = 8.5 \times 10^{-28}$	$t = -17.30$<br>$p = 1.1 \times 10^{-31}$
0.015	$t = -2.57$<br>$p = 1.1 \times 10^{-2}$	$t = -12.52$<br>$p = 4.0 \times 10^{-22}$	$t = -16.74$<br>$p = 1.2 \times 10^{-30}$
0.020	$t = -2.66$<br>$p = 9.0 \times 10^{-3}$	$t = -18.04$<br>$p = 4.6 \times 10^{-33}$	$t = -11.30$<br>$p = 1.70 \times 10^{-19}$
