# Supplementary material for: Communication Efficiency and Congestion of Signal Traffic in Large-Scale Brain Networks
Source: PLoS Comput Biol. 2014 Jan 9;10(1):e1003427. doi: 10.1371/journal.pcbi.1003427 (PMC3886893; doi:10.1371/journal.pcbi.1003427)
Supplement: Table S4 — Comparisons for the node contents statistic. The average of 100 simulations on the CoCoMac network was compared against 100 simulations on randomized networks, for 100 null network realizations. The entries represent nodes with statistically significant differences, and the average -statistics and -values for those 100 comparisons. (PDF) [file pcbi.1003427.s011.pdf]

node	t-statistic	p-value
PIT	$t = -10.95$	$p = 4.1 \times 10^{-22}$
CA1	$t = 34.25$	$p = 3.9 \times 10^{-85}$
23c	$t = 17.96$	$p = 1.9 \times 10^{-43}$
24	$t = -3.04$	$p = 2.7 \times 10^{-3}$
31	$t = 14.29$	$p = 2.7 \times 10^{-32}$
LIP	$t = -11.31$	$p = 3.2 \times 10^{-23}$
7b	$t = -5.92$	$p = 1.4 \times 10^{-8}$
8A	$t = -10.59$	$p = 4.6 \times 10^{-21}$
46	$t = -10.81$	$p = 1.1 \times 10^{-21}$
32	$t = 15.83$	$p = 5.2 \times 10^{-37}$
13a	$t = 23.73$	$p = 8.4 \times 10^{-60}$
