# Supplementary material for: Communication Efficiency and Congestion of Signal Traffic in Large-Scale Brain Networks
Source: PLoS Comput Biol. 2014 Jan 9;10(1):e1003427. doi: 10.1371/journal.pcbi.1003427 (PMC3886893; doi:10.1371/journal.pcbi.1003427)
Supplement: Text S1 — Supporting information text. The supporting information contains the following: explanation of the biological meaning of various model components (Section 1), an analytical model (Section 2), exploration of parameter space (Section 3), a statistical assessment of the similarity between network scenarios (Section 4), exact T- and P-values (Section 5), assessment of the effect of resampling simulation time series (Section 6) and an assessment of the effect of rich club size (Section 7). (PDF) [file pcbi.1003427.s012.pdf]

Supporting Material and Methods for:

Communication efficiency and congestion of signal traffic in large-scale brain networks

Bratislav Mišić*†‡ Olaf Sporns§ Anthony R. McIntosh†‡

*Corresponding Author: Bratislav Mišić, Rotman Research Institute, Baycrest, 3560 Bathurst Street, Toronto, ON, Canada, M6A 2E1 bratislav.misic@utoronto.ca

†Rotman Research Institute, Baycrest Centre, Toronto, Canada

‡Department of Psychology, University of Toronto, Canada

§Department of Psychological and Brain Sciences, Indiana University, Bloomington, Indiana, USA

1 Components of the model

The queueing network model used in the present study is relatively abstract and simple. Despite their long history of use in cognitive psychology [14], queueing models are not as physiologically realistic as other types of models. For example, nonlinear dynamic models can capture the physiological complexity of neural activity to a much greater extent [5, 6, 10, 11].

However, the purpose of our queueing model is not to re-create neural activity. Rather, it is intended as an analysis; a way to generate relative metrics about network communication, so that we can compare different network topologies to each other, or different nodes to each other. Our approach is similar to how graph theory is used in neuroscience, where networks can be compared on the basis of their efficiency, or brain regions can be compared on the basis of their centrality. By sacrificing some physiological realism, we are able to explicitly operationalize several aspects of network communication that could not otherwise be modeled by traditional means, such speed, throughput and fidelity. These metrics are not accessible with other types of models, such as nonlinear dynamic systems.

1.1 Discrete signal units

Central to our approach is the use of discrete signal units. Our primary interest is how pairs of nodes communicate in the context of the whole network, and these signal units merely represent the ability of brain regions to influence one another. This is a major assumption, because the diverse array of emergent neural phenomena, such as perception, cognition and motor control is surely more than just "message passing" between brain areas. However, this simplifying assumption allows us to trace the trajectory of each signal unit as it propagates in the network, and to calculate various metrics about the potential for communication that is afforded by the anatomical connectivity.

At the present time, there is no consensus in the literature as to what the basic unit of information is at this "large" spatial scale. The discrete packets in our model merely represent the ability of distal regions to influence each other. This is an assumption made by virtually every study in which the brain is represented as a large-scale graph.

1.2 Queues and buffers

The second major feature of our approach is the use of queues and finite buffers, which allow us to model how network topology constrains informa-

tion flow. A central tenet of cognitive psychology is the notion that we have limited cognitive resources, and this is reflected by a finite capacity for various faculties, such as attention and working memory. For instance, iconic memory is fleeting, barely lasting more than a few moments [21]. A typical high-functioning adult is only able to maintain approximately seven items in working memory [16]. Even the ability to simply register two successive events presents considerable difficulties and eventually becomes impossible if they occur within a short span of each other [18]. However, the reasons for such limits are unknown and typically attributed to scarce "cognitive resources".

Queueing is a mechanism by which signal units are made to interact as they flow through the network. Unlike graph theoretic models, a queueing network explicitly models the interplay between multiple information flows on top of a given topology. For example, a common graph-theoretic measure of node centrality is betweenness, calculated as the proportion of all shortest paths in the network that pass through a given node. This metric implicitly assumes that (a) information travels with perfect knowledge of global topology and (b) information travels completely unobstructed through the network. Thus, in a graph theoretic analysis, a node that is well-connected is assumed to be a hub. In a queueing network model, a node can be a hub or a bottleneck, and the framework allows us to investigate how the architecture of the network shapes and limits communication.

Likewise, finite buffers provide a way to model limited cognitive resources and imperfect information transmission in brain networks. Finite buffers allow for the possibility of signal loss, mimicking the poor fidelity of neural networks [7].

1.3 Poisson arrivals

"External" arrivals represent the assumption that new information is continuously generated and communicated in the network. The source of this information may be either stimulation exogenous to the nervous system, or some endogenous process. We chose to use Poisson arrivals for empirical reasons. In the single-cell literature, inter-spike intervals (ISIs) are thought to be exponentially distributed [13, 19, 20, 25]. Likewise, in the psychophysics and signal detection literature, the Poisson process is often used to model stimulus fluctuations and other statistical properties of the sensory environment, most notably in the classic models of Barlow [1] and McGill [15]. This assumption is largely based on the theoretical work of Pirenne [17], who showed that the number of light quanta impinging on the retina would have to be Poisson distributed, both temporally and spatially.

2 Analytical model

In the following section we seek to confirm the numerical model via analysis. We attempt to derive an analytical queueing network model comprised of N nodes. The network is represented as a binary adjacency matrix R of size $N \times N$. Each element $R_{i,j}$ is equal to 1 if there is a link from node i to node j. If there is no link from node i to node j then $R_{i,j} = 0$.

Each node is modeled as server which has to process a received signal unit before it is forwarded to a neighboring node. The routing of signal units is random, i.e. the signal units are forwarded to randomly selected neighbours.

The external arrivals to each node are modeled as a Poisson process. We assume that the sum of processing time at the intermediate node j and its forwarding time is exponentially distributed with parameter μ_j. In addition to biological considerations, we choose the exponential distribution because allows for a tractable analytical model and because it has a coefficient of variation equal to 1. The relatively conservative coefficient of variation allows for tighter performance bounds for many other sub-exponential service and forwarding times. We also assume that each node has a finite buffer and uses an impartial Last Come First Served (LCFS) service discipline with pushout. Thus, in the case of buffer overflow due to high input load, the signal unit that arrived least recently will be lost.

2.1 Modeling a node

In the classical M/M/1/K First Come First Served (FCFS) system with finite capacity, each arriving signal which finds the system (buffer and server) full is blocked and lost [12, 24]. Alternatively, in the pushout model, the arriving signal is always accepted, while the signal which has been waiting for the longest time is pushed out of the system. In this non-preemptive system, a signal which is already in service cannot be pushed out. It can be shown that the blocking probability of the wider class of M/G/1/K FCFS systems is equal to the pushout probability of a pushout system [24]. We will denote this value as P_B.

In addition, the probability distribution of buffer occupancy is the same for blocking and pushout systems as long as the service policy is impartial. However, it is also known [24] that the mean waiting time of a signal which is eventually served in LCFS pushout system is smaller than the waiting time in a FCFS system (although its variance is larger) [24].

Let us denote total arrival rate to node j as $\lambda_{in,j}$. Then offered load for

node is denoted as:

$$\rho_{o,j} = \frac{\lambda_{in,j}}{\mu_j}. \tag{1}$$

Then, probability of having $0 \leq k \leq K$ messages in the node's j buffer is given by:

$$p_{j,k} = \rho_{o,j}^k \frac{1-\rho_{o,j}}{1-\rho_{o,j}^{K+1}}. \tag{2}$$

Pushout, i.e. blocking probability is then equal to:

$$P_{j,B} = p_{j,K} = \rho_{o,j}^K \frac{1-\rho_{o,j}}{1-\rho_{o,j}^{K+1}}. \tag{3}$$

2.2 Modeling a network of nodes

According to Burke's theorem, the output of a M/M/m system is also a Poisson process [12]. This result can be extended to finite M/M/m/K systems due to the memoryless property of the distribution of signal interarrival times, which is exponential. This result allows for the analysis of any feedforward interconnection of nodes using node-by-node decomposition analysis.

Likewise, Jackson's theorem extends the previous result to a network of N nodes with feedback interconnections and allows a node-by-node decomposition [12]. This fits our analysis since the queuing network based on the macaque anatomy has a significant feedback structure.

Let us define P_{dest} as the probability that the node which receives the signal unit is a destination for that unit. We also define the probability that the node which receives the unit has to forward it further as $P_{tr} = 1 - P_{dest}$, i.e. the transit probability. Under the random routing assumption, these probabilities depend on the topology of the graph. A conservative estimate would be $P_{tr} = \frac{1}{N-1}$ and $P_{dest} = 1 - \frac{1}{N-1}$, although we will discuss less conservative approaches as well.

Let us assume that each node j in the network has an external input Poisson rate γ_j. The remaining part of the input rate comes from nodes that project to node j. By Burke's Theorem, the output process of each node is also Poisson [12].

Under the decomposition approach, the input Poisson rate to node j is equal to:

$$\lambda_{in,j} = \gamma_j + \sum_{i=1}^{N} \lambda_{out,i} r_{i,j} \tag{4}$$

where $r_{i,j}$ denote routing probabilities from node i towards node j. Under random routing and a known connectitivity matrix R, routing probabilities are equal to:

$$r_{i,j} = \frac{R_{i,j}}{od_l}, \tag{5}$$

where $od_i = \sum_{n=1}^{N} R_{i,n}$ is the output degree of node i.

The output rate from each node is affected by blocking caused by the node's finite buffer and by the portion of signals for which this node is a destination. Therefore, the output rate from node j is equal to:

$$\lambda_{out,j} = \lambda_{in,j}(1 - P_{j,B})P_{tr} \tag{6}$$

note that the output rate is also known as the throughput of the node. The carried load for node j can also be obtained as $\rho_{c,j} = \rho_{o,j}(1 - P_{j,B})P_{tr}$.

Equations (3), (4) and (6) for each node form a system of $3N$ equations which can be solved in an iterative way. The first iteration of the system is started with only external input to the nodes and iterations are stopped when the relative increment of blocking probabilities between two iterations falls below a threshold of 0.0001.

2.3 Modeling delay in a node

Let us first consider the FCFS system and the waiting time of the signal that arrives to the buffer and finds $k - 1 > 0$ waiting messages and one signal being serviced. That signal has to wait for remaining service time of the signal being served plus $k - 1$ complete service times.

Assume that the probability density function (pdf) of the message service time is denoted as $b_j(x) = \mu_j e^{-\mu_j x}$ and its probability distribution function (PDF) is $B_j(x) = \int_{y=0}^{x} b_j(y)dy$. The mean value of service time is $\bar{b} = \int_{x=0}^{\infty} xb(x)dx$.

The Laplace-Stieltjes Transform (LST) of message service time in node j is denoted as:

$$B_j^*(s) = \int_0^\infty e^{-sx} b_j(x)dx = \frac{\mu_j}{\mu_j + s} \tag{7}$$

The pdf of remaining service time is $b_{j,+}(x) = \frac{1-B_j(x)}{\bar{b}}$. The LST of the remaining signal service time is denoted as [4, 23]:

$$B_j^{+*}(s) = \int_{x=0}^\infty e^{-sx} b_{j,+}(x)dx = \frac{1 - B_j^*(s)}{s}. \tag{8}$$

Due to the memoryless property, for the exponential distribution it holds that $B_j^{+*}(s) = B_j^*(s) = \frac{\mu_j}{\mu_j+s}$.

Therefore, the LST of the delay in FCFS system with an exponentially distributed service time has the form:

$$
\begin{aligned}
W^*_{fcfs,j}(s) &= \frac{1}{1-P_{j,B}}\left(p_{j,0} + \sum_{k=1}^{K-1} p_{j,k} B_j^{+*}(s) B_j^*(s)^{k-1}\right) \\
&= \frac{1}{1-P_{j,B}}\left(p_{j,0} + \sum_{k=1}^{K-1} p_{j,k} B_j^*(s)^{k}\right) \qquad (9)
\end{aligned}
$$

The mean value of the delay can be derived as $\overline{W^*_{fcfs,j}} = -\left.\frac{dW^*_{fcfs,j}(s)}{ds}\right|_{s=0}$.

Analysis of message delay in the LCFS system with pushout is more involved due to the recursive nature of the delay process. In order to derive a distribution of this delay we first attain some intermediate results.

First, we define the LST of the joint distribution of service time and number of signals that arrive during service time as $a^*_{j,n}$. By the same token, we define the LST of the joint distribution of residual signal service time and number of signal arrivals as $d^*_{j,n}$. Expressions for $a^*_{j,n}$ and $d^*_{j,n}$ are presented as:

$$
a^*_{j,n}(s) = \int_{x=0}^{\infty} \frac{(\lambda_{in,j}x)^n}{n!} e^{-(s+\lambda_{in,j})x} b(x)dx \qquad (10)
$$

$$
d^*_{j,n}(s) = \int_{x=0}^{\infty} \frac{(\lambda_{in,j}x)^n}{n!} e^{-(s+\lambda_{in,j})x} b_{j,+}(x)dx \qquad (11)
$$

Note that for the exponential distribution of service time $a^*_{j,n}(s) = d^*_{j,n}(s)$. We also denote $W^*_{j,k}(s)$ as the LST of the delay for the waiting time of a signal that has k signals ahead at the end of service and is eventally served (index k ranges from $0 \le k \le K-2$).

$$
\begin{aligned}
W^*_{j,0}(0) &= 0 \\
W^*_{j,k}(s) &= \sum_{n=0}^{K-k-1} a^*_{j,n}(s) W^*_{j,k+n-1}(s), \quad 1 \le k \le K-2 \qquad (12)
\end{aligned}
$$

Using elimination, the system of equations (12) can be solved such that $W^*_{j,k}(s) = f(a^*_{j,n}(s)),\ \ n = 0..K-2$.

For signals that receive service, the LST of the delay has been presented as [24]:

$$W^*_{lcfs,j}(s) = \pi_{j,0} + \rho_{o,j} \sum_{k=0}^{K-2} a^*_{j,k}(s) W^*_{j,k}(s) \quad (13)$$

where $\pi_{j,0}$ denotes the probability that the system is empty at signal departure time. Fortunately, for exponentially distributed service times $\pi_{j,0} = p_{j,0}$.

The mean value of node delay can be obtained as $\overline{W^*_{lcfs,j}} = -\left.\frac{dW^*_{lcfs,j}(s)}{ds}\right|_{s=0}$.

Finally, we need to mention that the signal response time for node j is the sum of signal waiting and processing times, i.e. $T^*_{lcfs,j}(s) = W^*_{lcfs,j}(s)B^*(s)$ and $\overline{T^*_{lcfs,j}} = \overline{W^*_{lcfs,j}} + \frac{1}{\mu}$.

2.4 End to end delay (transit times)

Given the fact that CoCoMac graph is relatively large, well connected and that we use random routing, we used a computationally efficient approximation, whereby the number of traversed hops between source and destination node has a probability distribution similar to the geometric [8]. However, a parameter of this distribution is the transit probability P_{tr}, which can not be fully oblivious to the traversed path. If it was fully oblivious to the traversed path, then $P_{tr} = \frac{1}{N-1}$, giving the Probability Generating Function (PGF) of the total number of hops:

$$N_{hp}(z) = \sum_{k=0}^{\infty} P^k_{tr}(1 - P_{tr}) z^{k+1} \quad (14)$$

giving mean number of hops as $\overline{N_{hp}} = \left.\frac{dN_{hp}(z)}{dz}\right|_{z=1} = \frac{1}{1-P_{tr}} = N - 1$.

A better approximation would assume that the transit probability decreases with each traversed hop until the number of hops reaches a number that is significantly larger than the diameter of the network. In the case of the macaque network with diameter equal to 4, we limit this to $N - 1$, which is 60 times larger than the diameter. In that case, for i traversed hops $P_{tr,i} = \frac{1}{N-i}$, and the PGF for the number of traversed hops becomes:

$$N_{hp,m}(z) = \sum_{k=0}^{N} \prod_{i=1}^{k} P_{tr,i}(1 - P_{tr,k+1}) z^{k+1} \quad (15)$$

The mean number of hops using this approximation is approximately $N/2$.

The LST of the end to end delay and processing time for signals originating in node j - according to both approaches - is:

$$E_j^*(s) = \sum_{k=0}^{\infty} P_{tr}^k (1 - P_{tr}) T_{lcfs,j}^*(s)^{k+1} \tag{16}$$

$$Em_j^*(s) = \sum_{k=0}^{\infty} \prod_{i=1}^{k} P_{tr,i}(1 - P_{tr,k+1}) T_{lcfs,j}^*(s)^{k+1} \tag{17}$$

with mean values of $\overline{E_j} = (N-1)\overline{T_{lcfs,j}^*}$ and $\overline{Em_j} = -\left.\frac{dEm_j^*(s)}{ds}\right|_{s=0}$.

Additional averaging over all nodes in the graph gives $\overline{E} = \sum_{j=1}^{N} \overline{E_j}/N$.

2.5 Comparing analysis and simulation

As mutual verification of the analytic expressions derived above and the numerical simulations reported in the main manuscript, we compare the two for network- and node-level statistics (Figs. S3,S4). In general, the two methods are in good agreement for the node-level statistics (Fig. S4). There is also considerable agreement for the network-level statistics (Fig. S3) and the two methods show the same pattern of results. There are also some small differences between the two, particularly for transit times, and these are likely due to the fact that we had to approximate the distribution of “hops” from source to target node using the geometric distribution.

3 Exploring parameter space

In general, the present model has two characteristic modes of operation. At low intensities (external arrival rates) the total number of signal units in the network fluctuates around some finite value and the system is stable (Fig. 2). As the intensity is increased, there is a qualitative change in the system dynamics, characterized by a monotonic increase in the number of signal units in the network until all buffers are filled. This is analogous to a phase transition in dynamical systems theory and is sometimes referred to as a “jamming” transition [2, 22]. The focus of the present study was on the steady-state behavior of the network.

The key variable is the ratio between the arrival rate and service rate at each node. Therefore, we fixed the service rate (μ) and varied the rate of external arrivals (λ). The latter effectively becomes an order parameter, capable of inducing a phase transition at some critical value.

The only other free parameter is the buffer capacity H. Buffer capacity is not a critical parameter in the sense that it cannot induce a phase transition in the system. Changes in buffer capacity will produce quantitative, but not qualitative, changes in system behaviour. To demonstrate this point, we show node-specific metrics at three different capacities in Fig. S5. As buffer capacity is increased from $H = 5$ to $H = 100$, the total number of signal units in the system will increase because congested nodes can now hold more signal units in their buffers. Node contents are increased, particularly for the high-degree rich club nodes. Likewise, blocking probabilities remain unchanged, save for a few nodes where, due to the higher capacities, fewer signal units are lost. Node utilizations are slightly increased for all nodes, because fewer signal units are lost and there is a greater number of signal units in the system. However, the utilizations are fairly consistent across different buffer capacities, because the limiting factor in the utilization of each node is the ratio of arrival rate and service rate at each node.

4 Assessing similarity between network scenarios

To assess the similarity between the patterns of results produced by the three different scenarios (CoCoMac, Small-World and Rich-Club), we employed the following procedure. For each network measure (e.g. transit time), we constructed a vector containing the results for the original, randomized and latticized networks at all simulation intensities (0.05, 0.1, 0.15, 0.2). For instance, for the CoCoMac (C) network and its randomized (R) and latticized (L) null models, the vector was organized as:

$$V_{CoCoMac} = \begin{pmatrix} C(0.05) \\ R(0.05) \\ L(0.05) \\ \vdots \\ C(0.20) \\ R(0.20) \\ L(0.20) \end{pmatrix} \tag{18}$$

This vector, representing the overall pattern of results for a given measure, was then correlated with analogous vectors constructed for the Small-World and Rich-Club scenarios. The correlation coefficients and associated p-values are displayed in Table S1. Both the Small-World and Rich-Club scenarios are highly correlated with the CoCoMac pattern and this is due to the fact

that increasing simulation intensity leads to similar behaviour in all networks, such as increasing throughput, blocking and utilization. Importantly, patterns associated with the CoCoMac scenario were consistently more highly correlated with the Rich-Club scenario (≈ 0.99) than with the Small-World scenario (≈ 0.85). Note that these results are invariant to whether the vector is constructed by arranging networks within intensities (as shown above) or intensities within networks.

Finally, to assess whether there is any difference between the correlation coefficients obtained for CoCoMac-Small World and CoCoMac-Rich Club scenarios, we apply Fisher's r-to-z transformation. Following Cohen and Cohen [3], we express the difference between the two transformed correlation coefficients as a z score (Table S1, bottom row). The comparison is treated as a one-tailed test, and z scores greater than $|1.645|$ are taken to indicate that the CoCoMac-Rich Club correlation is significantly greater than the CoCoMac-Small World correlation.

5 T-values and p-values

In section 2.1 of the main manuscript we report differences in network and node statistics for the CoCoMac network and it's degree-matched randomized and latticized null models. Statistical assessment was performed by taking the average results for 100 simulations on the CoCoMac network and comparing these to 100 simulations on 100 realizations of the null networks. The average t-statistics and p-values associated with these comparisons are shown in Tables S2 (transit times), S3 (throughput) and S4 (node contents). Thus, Tables S2 and S3 correspond to Fig. 2 in the main manuscript, while Table S4 corresponds to Fig. 4 in the main manuscript.

6 Effect of resampling

In a discrete-event simulation, the system state is updated only when an event occurs. This event may be the creation of a signal unit, a signal unit entering a queue or leaving a server, or a signal unit being removed from the network. As a result, in many models system state is sampled at non-uniform time intervals. This is true of the present model, because system dynamics are governed by random variables, such as signal inter-arrival times and service times.

To facilitate conventional time series analysis, we used simple linear interpolation (also known as "table lookup") to resample the system state at

uniform time intervals. The new time series were effectively downsampled because an original simulation run had an average of 2.21 samples per time unit, while the interpolated time series were sampled once per time unit.

Fig. S6 shows the effects of linear interpolation for one sample time series of network load. Compared to the original (Fig. S6), the interpolated time series appears slightly smoother (Fig. S6B), indicating some loss of high frequencies as expected. However, there appears to be little difference between the two time series.

7 Effect of rich club size

To demonstrate that network-level behavior reported in the manuscript could be replicated with a wide range of synthetic rich club networks, we create three additional such networks, with rich clubs of 10, 20 and 30 nodes. As described in the main manuscript, the networks were created from a random network by endowing a sub-set of nodes (the rich club) with greater connection density than the rest of the network, and an even greater connection density amongst each other.

Fig. S7 shows the network-level metrics for each of these synthetic rich club networks, as well as their randomized and latticized versions. The pattern of results appears to be invariant to rich club size, and very similar to the network with 25 rich club nodes reported in the main manuscript. The small differences between the networks are likely due to the slight differences in density between the networks.

References

[1] Barlow, H. (1956). Retinal noise and absolute threshold. *J Opt Soc USA*, 46(8):634–639.

[2] Boccaletti, S., Latora, V., Moreno, Y., Chavez, M., and Hwang, D. (2006). Complex networks: Structure and dynamics. *Phys Rep*, 424(4):175–308.

[3] Cohen, J. and Cohen, P. (1975). *Applied multiple regression/correlation analysis for the behavioral sciences.* Lawrence Erlbaum.

[4] Cooper, R. B. (1981). *Introduction to Queueing Theory.* North Holland, New York, 2nd edition.

[5] Deco, G., Jirsa, V., and McIntosh, A. (2011). Emerging concepts for the dynamical organization of resting-state activity in the brain. *Nat Rev Neurosci*, 12:43–56.

[6] Deco, G., Jirsa, V., McIntosh, A., Sporns, O., and Kötter, R. (2009). Key role of coupling, delay, and noise in resting brain fluctuations. *Proc Natl Acad Sci USA*, 106(25):10302–10307.

[7] Faisal, A., Selen, L., and Wolpert, D. (2008). Noise in the nervous system. *Nat Rev Neurosci*, 9(4):292–303.

[8] Grimmett, G. R. and Stirzaker, D. R. (1992). *Probability and Random Processes.* Oxford University Press, Oxford, UK, 2nd edition.

[9] Harriger, L., van den Heuvel, M., and Sporns, O. (2012). Rich club organization of macaque cerebral cortex and its role in network communication. *PLoS ONE.*

[10] Honey, C., Kötter, R., Breakspear, M., and Sporns, O. (2007). Network structure of cerebral cortex shapes functional connectivity on multiple time scales. *Proc Natl Acad Sci USA*, 104(24):10240–10245.

[11] Jirsa, V., Sporns, O., Breakspear, M., Deco, G., and McIntosh, A. (2010). Towards the virtual brain: network modeling of the intact and the damaged brain. *Arch Ital Biol*, 148:189–205.

[12] Kleinrock, L. (1972). *Queuing Systems*, volume I: Theory. John Wiley and Sons, New York.

[13] Koch, C. and Segev, I. (1998). *Methods in Neuronal Modeling: From Ions to Netwoks*. The MIT Press.

[14] Liu, Y. (1996). Queueing network modeling of elementary mental processes. *Psychol Rev*, 103(1):116–136.

[15] McGill, W. (1967). Neural counting mechanisms and energy detection in audition. *J Math Psychol*, 4(3):351–376.

[16] Miller, G. (1956). The magical number seven, plus or minus two: Some limits on our capacity for processing information. *Psychol Rev*, 63:81–97.

[17] Pirenne, M. (1951). Quantum physics of vision: theoretical discussion. *Prog Biophys Biop Ch*, 2:192–223.

[18] Raymond, J., Shapiro, K., and Arnell, K. (1992). Temporary suppression of visual processing in an RSVP task: An attentional blink. *J Exp Psychol Human*, 18(3):849–860.

[19] Reich, D., Victor, J., and Knight, B. (1998). The power ratio and the interval map: spiking models and extracellular recordings. *J Neurosci*, 18(23):10090–10104.

[20] Rieke, F., Warland, D., De Ruyter van Steveninck, R., and Bialek, W. (1997). *Exploring the neural code*. The MIT press.

[21] Sperling, G. (1960). The information available in brief visual presentations. *Psychol Monogr*, 74(11):1–29.

[22] Tadić, B., Thurner, S., and Rodgers, G. (2004). Traffic on complex networks: Towards understanding global statistical properties from microscopic density fluctuations. *Phys Rev E*, 69(3):036102.

[23] Takagi, H. (1991). *Queueing Analysis*, volume 1: Vacation and Priority Systems. North-Holland, Amsterdam, The Netherlands.

[24] Takagi, H. (1993). *Queueing Analysis*, volume 2: Finite Systems. North-Holland, Amsterdam, The Netherlands.

[25] Tuckwell, H. (2005). *Introduction to theoretical neurobiology: Volume 2, nonlinear and stochastic theories*, volume 8. Cambridge University Press.
